# The Philosophy of the Moving Powers of the Blood

**Published:** 1845-04

**Authors:** 


ART. III.—*The Philosophy of the Moving Powers of the Blood.* By G. CALVERT HOLLAND, M.D. Physician extraordinary to the Sheffield Infirmary, &c. &c.—*London*, 1844. 8vo, pp. 308.

THIS volume consists of a series of essays on the circulation, which have from time to time appeared in the 'Edinburgh Medical and Surgical Journal,' and which have been the result of long-continued attention to the subject. Dr. Holland's object seems to have been rather to criticise the experimental results obtained by others, than to advance anything particularly new of his own; at least we cannot see that the results at which he has arrived, differ essentially from those of other physiologists, who agree with him in regarding the heart's action as by no means the sole moving power in the circulation. He exposes very clearly the fallacy of the arguments on the other side, which have been drawn from the experiments of Magendie, Poiseuille, Barry, &c., and points out the difficulty—nay, almost the impossibility—of devising experiments on this subject which shall be really conclusive, in consequence of the intimate relationship which exists amongst the different vital operations, causing any interference with *one* to give an unnatural condition to the *rest*, which must in turn react upon the first. Dr. Holland contents himself with demolishing the fortifications which his opponents have cast up around their theory; but he does not substitute anything very definite in its place. The work cannot be regarded, therefore, as a complete treatise upon the circulation; for there is scarcely any reference to comparative anatomy, and none to embryology, in both of which departments numerous valuable results of "experiments prepared by nature" are to be met with. Our own views in regard to the presence, in the capillary circulation, of a certain force independent of the heart's action, have been frequently expressed; and we need not, therefore, discuss the subject over again, but shall simply recommend Dr. Holland's work to the attention of such of our readers as wish to go more deeply into the question, than they can do by means of the ordinary treatises on physiology.

